# The Intracellular Bacterium *Wolbachia* Uses Parasitoid Wasps as Phoretic Vectors for Efficient Horizontal Transmission

**DOI:** 10.1371/journal.ppat.1004672

**Published:** 2015-02-12

**Authors:** Muhammad Z. Ahmed, Shao-Jian Li, Xia Xue, Xiang-Jie Yin, Shun-Xiang Ren, Francis M. Jiggins, Jaco M. Greeff, Bao-Li Qiu

**Affiliations:** 1 Department of Entomology, South China Agricultural University, Guangzhou, China; 2 Department of Genetics, University of Pretoria, Pretoria, South Africa; 3 Department of Genetics, University of Cambridge, Cambridge, United Kingdom; University of Liverpool, UNITED KINGDOM

## Abstract

Facultative bacterial endosymbionts are associated with many arthropods and are primarily transmitted vertically from mother to offspring. However, phylogenetic affiliations suggest that horizontal transmission must also occur. Such horizontal transfer can have important biological and agricultural consequences when endosymbionts increase host fitness. So far horizontal transmission is considered rare and has been difficult to document. Here, we use fluorescence in situ hybridization (FISH) and multi locus sequence typing (MLST) to reveal a potentially common pathway of horizontal transmission of endosymbionts via parasitoids of insects. We illustrate that the mouthparts and ovipositors of an aphelinid parasitoid become contaminated with *Wolbachia* when this wasp feeds on or probes *Wolbachia*-infected *Bemisia tabaci* AsiaII7, and non-lethal probing of uninfected *B. tabaci* AsiaII7 nymphs by parasitoids carrying *Wolbachia* resulted in newly and stably infected *B. tabaci* matrilines. After they were exposed to infected whitefly, the parasitoids were able to transmit *Wolbachia* efficiently for the following 48 h. Whitefly infected with *Wolbachia* by parasitoids had increased survival and reduced development times. Overall, our study provides evidence for the horizontal transmission of *Wolbachia* between insect hosts by parasitic wasps, and the enhanced survival and reproductive abilities of insect hosts may adversely affect biological control programs.

## Author Summary

Vertically-transmitted facultative bacterial endosymbionts are common in invertebrates, and affect traits as diverse as the mode of sexual reproduction, speciation, and susceptibility to pathogens. Horizontal transmission of endosymbionts is thought to be infrequent in most species, and not to contribute to their spread through populations. Here we demonstrate that parasitoid wasps can act as vectors, transmitting the endosymbiont *Wolbachia* between whitefly hosts at a high rate. The ovipositors and mandibles of parasitoids can be contaminated with *Wolbachia* when probing infected whitefly. If these parasitoids then probe *Wolbachia*-free hosts and the whitefly survive, it will result in a stably infected line with increased fitness. Such vector-borne transmission may explain why endosymbionts are so widely distributed, and why genetically similar symbionts are often found in phylogenetically distant organisms.

## Introduction

Vertically transmitted intracellular bacteria often live in symbioses with their arthropod hosts [1]. These endosymbionts may be obligate (essential for host survival) or facultative, in which case they can increase or decrease host fitness [1–3]. When endosymbionts are obligate they typically share a long evolutionary history with their hosts and are found within specialized cells [4]. The facultative endosymbionts tend to have a more recent association with arthropods, but are nonetheless very common among arthropods. For instance, 40% of insect species are estimated to be infected with *Wolbachia* [5]. Similar to obligate endosymbionts, facultative endosymbionts are also transmitted vertically from mother to offspring with high fidelity and this is considered the primary transmission pathway [4]. However, through their evolution these endosymbionts have also been transmitted horizontally between different species [1,6,7], with closely related endosymbionts occurring in phylogenetically distant insects [1,7–12]. In the last two decades there have been multiple studies reporting evidence of *Wolbachia* transmission within and between both phylogenetically close and more distant species either through phylogenetic or transinfection studies [8,13–18].

The spread of endosymbionts in field populations by horizontal transmission, however, has received comparatively little attention, and the mechanisms driving horizontal transmission are only recently becoming apparent [1,3]. Horizontal transmission of symbionts has been documented when infected and uninfected parasitoid wasps develop within the same host insect [19–21], when infected males mate with uninfected females [22], when infected and uninfected whiteflies feed on the same host plant and the symbiont moves through the phloem sap [23], and when symbionts are acquired from the environment [24]. It is also possible for endosymbionts to be transmitted by vectors. *Hamiltonella defensa* and *Regiella insecticola* symbionts can be efficiently transmitted when a parasitoid wasp sequentially stabs an infected then an uninfected aphid [25]. Similarly, *Spiroplasma* can be transmitted between *Drosophila* species by ectoparasitic mites [26]. Here we report the efficient phoretic transfer of *Wolbachia* by the parastoid *Eretmocerus* sp. nr. *furuhashii* from infected whitefly *Bemisia tabaci* AsiaII7 to uninfected individuals..

The whitefly *Bemisia tabaci* is a small hemipterous insect that feeds on phloem sap of numerous host plants. It is currently considered as a complex of at least 24 distinct cryptic species that are morphologically indistinguishable but markedly differ in host range, ability to transmit viruses, insecticide resistance and the endosymbionts they are infected with [27–30]. The *B*. *tabaci* species complex harbours various bacterial symbionts, including *Wolbachia*. *Wolbachia* has been found in the ovarian cells of the host and at the circumference of and inside the bacteriocytes [31]. The AsiaII7 *B*. *tabaci* is indigenous to China and is one of the common cryptic species in South China (formerly “Cv” biotype) [32]. It was first discovered on variegated laurel *Codiaeum variegatum* in Guangzhou in 2006, and further studies indicated that this whitefly can damage various economically important ornamental plants [33]. *Eretmocerus* sp. nr. *furuhashii* is one of the dominant parasitoids of whitefly *B*. *tabaci* in South China [34,35]. It is a primary, solitary parasitoid which oviposits externally between the nymphal host and the leaf surface, but nonetheless penetrates whitefly nymphs with its mouth parts and ovipositor to examine them before laying and to feed.

The current study was motivated by a six-year long observation from 2007–2012 of two subcolonies, one for rearing whitefly *B*. *tabaci* AsiaII7 (hereafter “WR”, 5 cages) and the other for rearing the parasitoid *E*. sp. nr. *furuhashii* using AsiaII7 as hosts (hereafter “PR”, 6 cages). We found that the *Wolbachia* infection rate of whitefly housed with parasitoids was higher than that without parasitoids, gradually increasing during the surveillance period (S1 Fig.). Meanwhile, the prevalence of *Wolbachia* in *E*. sp. nr. *furuhashii* wasp was lower than that of the whitefly, but also gradually increased (S1 Fig.). Although this is not a replicated experiment, it led us to hypothesise that the coexistence of parasitoid and whitefly may promote the transmission of *Wolbachia* between different individuals of whitefly. Here we show that non-lethal host inspection of the whitefly nymphs by the wasp can transfer *Wolbachia*. The acquisition of *Wolbachia* benefited its host’s fitness in terms of faster immature development and increased adult survival.

## Results

### Behavioral observation of parasitoids visiting whitefly

To test whether parasitoids can vector *Wolbachia*, we allowed them to oviposit and feed on *Wolbachia-*infected whitefly and then transferred them to a cage of uninfected whitefly. By observing the behavior of *E*. sp. nr. *furuhashii* in the laboratory, we found that among the uninfected AsiaII7 nymphs which were visited by parasitoids for feeding or oviposition checking, 35.8% (38 of 106) died, adult parasitoids emerged from 34.0% (36 of 106), and 30.2% (32 of 106) resulted in adult whitefly. *Wolbachia* screening by PCR revealed that 93.8% (30 of 32) of the newly emerged whitefly individuals became infected after surviving the parasitoid penetration.

### FISH detection and MLST of *Wolbachia* in whitefly and parasitoids

**Fig 1:**
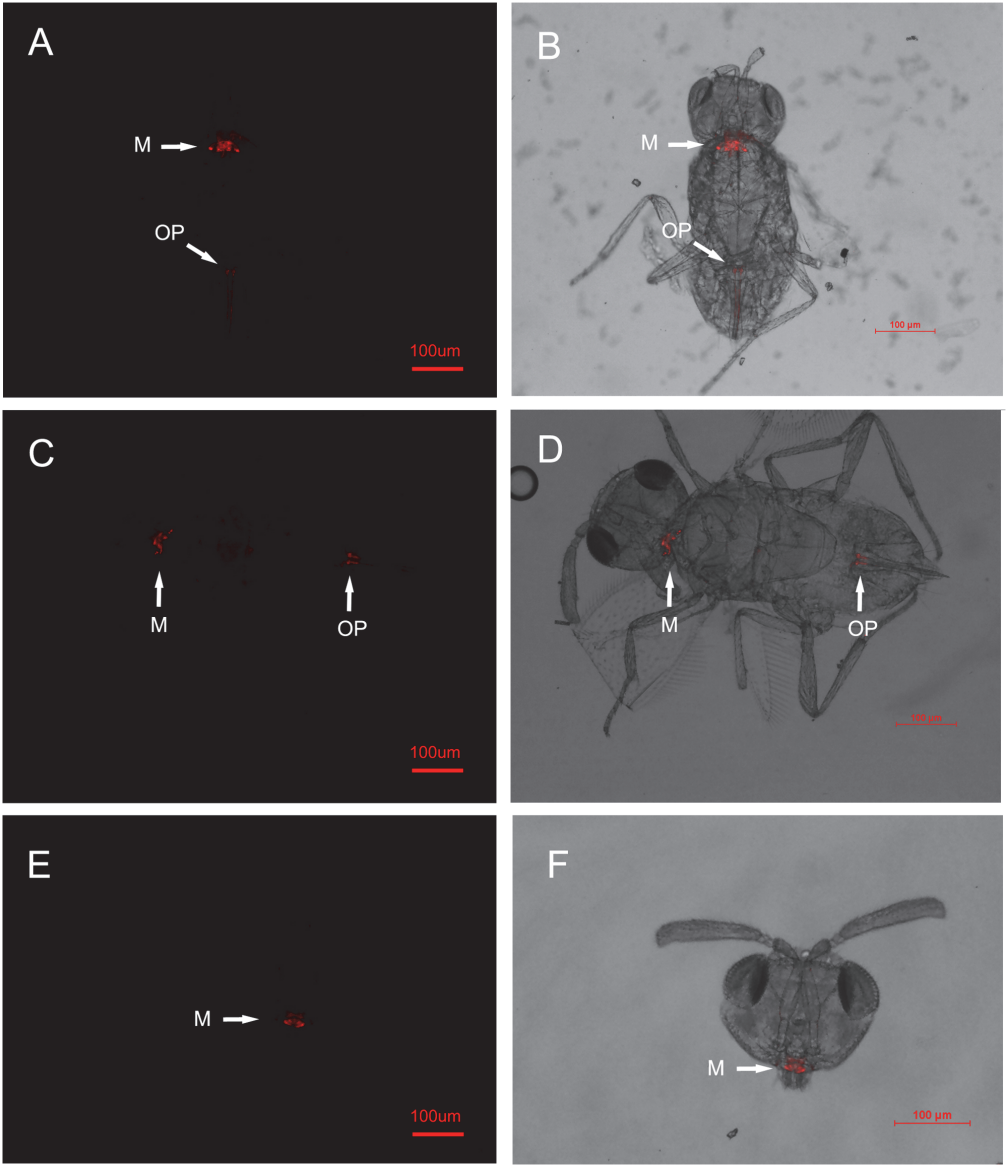
FISH detection of *Wolbachia* in adult *Eretmocerus* parasitoids. The *E*. sp. nr. *furuhashii* wasps were selected randomly 24–48 h after visiting *Wolbachia*-positive AsiaII7 whitefly nymphs. Panels (A-D): *Wolbachia* in parasitoid with different body poses, it was found both in the mouthparts (M) and ovipositors (OP); panels (E, F): *Wolbachia* in the parasitoid mouthparts (front view). Left panels (A, C, E): fluorescence in dark field; right panels (B, D, F): fluorescence in bright field.

**Fig 2:**
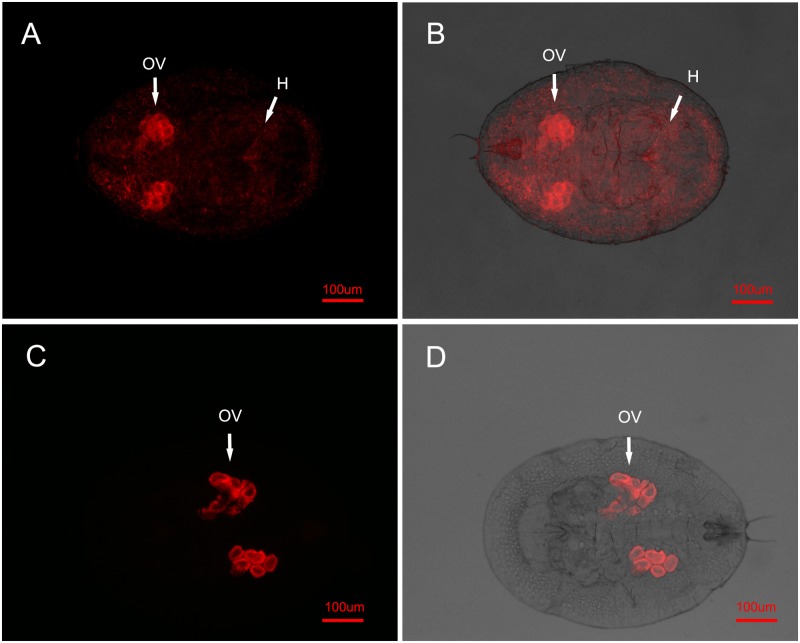
FISH detection of *Wolbachia* in AsiaII7 whitefly. *Wolbachia* has two distributions in 3^rd^ instar AsiaII7 nymphs randomly selected from the *Eretmocerus* rearing cages. Top panels (A, B): in the scattered pattern, *Wolbachia* was found both in the ovary (OV) and haemolymph (H) of whitefly; bottom panels (C, D): in the confined pattern, *Wolbachia* was only found in the ovary (OV) of whitefly. Left panels (A, C): fluorescence in dark field; right panels (B, D): fluorescence in bright field.

In order to confirm infection by *Wolbachia*, samples of both AsiaII7 nymphs (including the donor whitefly from PR cage, the newly infected nymphs and their offspring) and *E*. sp. nr. *furuhashii* were selected randomly for fluorescence in situ hybridization. We found different distributions of *Wolbachia* in the two-level trophic system. For the parasitoid *E*. sp. nr. *furuhashii*, *Wolbachia* was found both in their mouthparts and ovipositor after they fed on or penetrated whitefly hosts, but not in the ovaries (Fig. 1). Among the donor AsiaII7 nymphs there were two distinct patterns of infection. In some individuals *Wolbachia* was “scattered” in both the ovaries and somatic tissues (Fig. 2 A, B; 6 of 30 individuals), while in others it was “confined” to the bacteriocytes and ovaries (Fig. 2 C, D; 24 of 30 individuals).

In uninfected whitefly that had been visited by a contaminated parasitoid, *Wolbachia* was not visible during the first 72 h. This is presumably due to its low density, as qRT-PCR showed a continuous increase of *Wolbachia* during this time, indicating that the insect had been infected and the bacterium could replicate (S2 Fig.). In the offspring of whitefly visited by contaminated wasps, *Wolbachia* is clearly visible in 3^rd^ instar nymphs. In all cases the symbiont had the scattered pattern (15 of the 16 AsiaII7 offspring harboured scattered *Wolbachia*, while *Wolbachia* was not detected in the other one).

To identify the *Wolbachia* strain in AsiaII7 whiteflies and *Eretmocerus* parasitoids, we sequenced five MLST genes and the *wsp* gene. This revealed that the *Wolbachia* strain from *E*. sp. nr. *furuhashii* was the same as in AsiaII7 (sequence type 388). By comparing the sequences those in the *Wolbachia* MLST database (http://pubmlst.org/wolbachia/) and by constructing phylogeny, this was identified as a new sequence type “ST388” (S1 Table, S3 Fig.).

### Persistence of *Wolbachia* in whitefly and parasitoids

**Fig 3:**
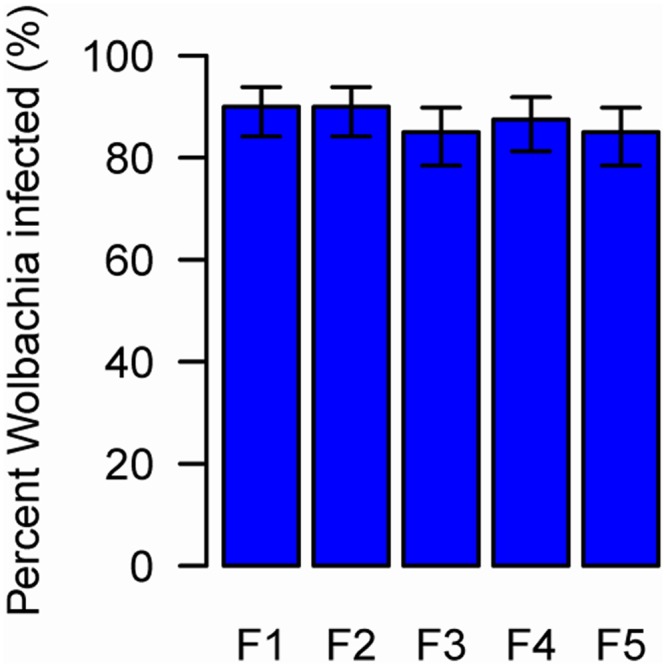
The prevalence of newly-acquired *Wolbachia* in AsiaII7 whitefly over five generations. The proportion on infected whitefly was measured in a population over 5 generations. The population was founded by four pairs of new emerged AsiaII7 whitefly that had been infected with *Wolbachia* acquired from a contaminated wasp. The proportion of *Wolbachia*-infected offspring was monitored with PCR. The bars are standard errors. The means and standard errors were estimated using a generalized linear model and are back-transformed from the logit scale. The is no significant heterogeneity in the prevalence among generations (GLMM likelihood ratio test: χ^2^ = 0.92, df = 4, *P* = 0.92).

To be stably transmitted between generations *Wolbachia* must infect the female germ line. We established populations of whitefly from individuals that had been probed by contaminated parasitoids and measured the prevalence of *Wolbachia* for five generations. We found that 85.0 to 90.0% of the F1 to F5 progeny of the newly-infected whiteflies were *Wolbachia*-positive (Fig. 3), indicating the vertical transmission and long term persistence of *Wolbachia* in the newly-infected AsiaII7 populations. While we did not directly measure vertical transmission rates (uninfected whitefly may have been among the parents of each generation), these results indicate efficient transmission from parent to offspring. The negative control was uninfected in all 5 generations.

**Fig 4:**
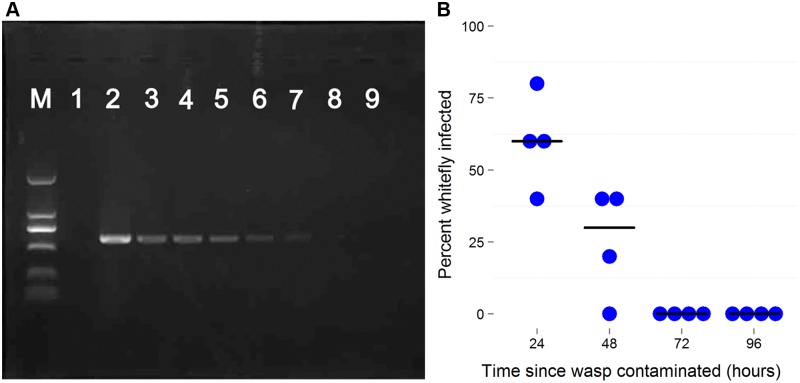
The persistence of newly-acquired *Wolbachia* in parasitoids. A: *Wolbachia* detection by PCR using *wsp* gene in the seven days after the parasitoid visited an infected host. In each PCR reaction *Wolbachia* DNA was extracted from 10 individuals. B: The decline in *Wolbachia* transmission rates with time after the wasp is contaminated. Female *Eretmocerus* wasps were released to parasitize *Wolbachia* negative AsiaII7 2^nd^-3^rd^ instar nymphs 24, 48, 72 and 96 h after contamination with *Wolbachia*. The *Wolbachia* infection rate of whitefly that were visited by wasps but survived to adulthood was detected with PCR. Each point is the transmission rate to ten whiteflies in a single replicate of the experiment. The horizontal bars are the median. There is a significant decline in transmission rates (logistic generalized linear model: *z* = 3.50, *P* = 0.0005).

*Wolbachia* DNA can be detected by PCR for at least 5 days in *E*. sp. nr. *furuhashii* after they fed or oviposited on *Wolbachia*-infected AsiaII7 nymphs (Fig. 4A). However, parasitoids contaminated with *Wolbachia* were only able to transmit it for the first 48 h, indicating that the bacteria are gradually losing their infectivity with time (Fig. 4B).

### Effects of *Wolbachia* on whitefly fitness

**Fig 5:**
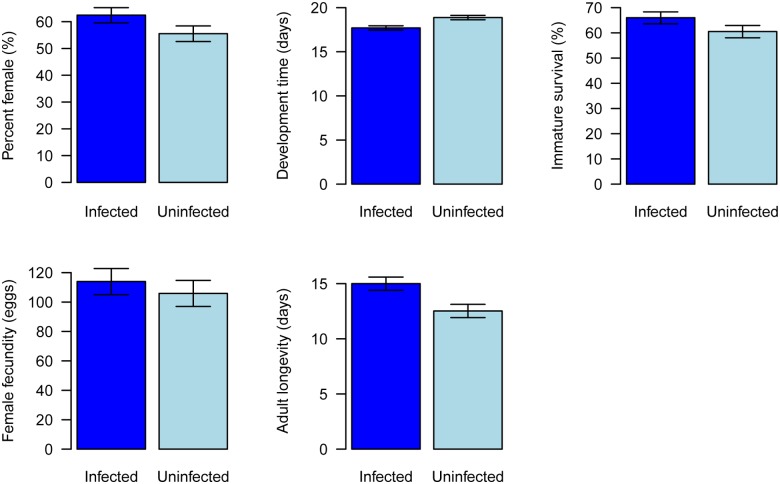
Effects of *Wolbachia* on whitefly fitness. Traits affecting the fitness of newly *Wolbachia*-infected offspring were compared with those *Wolbachia* uninfected populations under laboratory conditions. A: percent female, B: development time, C: immature survival, D: female adult fecundity and E: female adult longevity. The data are means ± SE.

We established populations of whitefly with and without *Wolbachia*, and found that they had significant biological differences. Compared to uninfected AsiaII7, infected whiteflies developed significantly faster (Fig. 5, General Linear Mixed Model: *t* = 3.38, df = 6, *P* = 0.01) and had significantly increased longevity (Fig. 5, Cox proportional hazards mixed model: *z* = 3.86, *P* = 0.0001). There was no significant effect of *Wolbachia* on either juvenile survival (Fig. 5, Generalised linear mixed model: *z* = 1.6, *P* = 0.11) or fecundity (Fig. 5, General Linear Mixed Model: *t =* 0.64, df = 6, *P* = 0.55). *Wolbachia* was associated with a slightly higher proportion of female offspring, but this was not significant (Fig. 5, Generalised linear mixed model: *z* = 1.7, *P* = 0.09).

### Effects of horizontal transmission on the population dynamics of *Wolbachia*

To understand how horizontal transmission will affect the prevalence of a symbiont, we simulated the spread of *Wolbachia* through a population. To do this we modified existing models of *Wolbachia* dynamics to include an additional parameter *w*, which is the product of the number of new cases generated by horizontal transmission from a single infected host in an otherwise uninfected population and any reduction in vertical transmission efficiency of *Wolbachia* in the newly infected hosts relative to those infected from their mother. The value of *w* will be determined by factors including the frequency with which a vector visit hosts, the time that contaminated vectors remain infectious, and the probability that a contaminated vector infects an uninfected whitefly. Field data shows that the majority of wasps are contaminated by *Wolbachia* DNA (S4 Fig.), and our lab data suggests many of these will be infectious (DNA is detectable for 5 days, the infectious period is 2 days). We also find that about a third of nymphs survive parasitism. Therefore, *w* will be below the parasitism rate, but not dramatically so.

**Fig 6:**
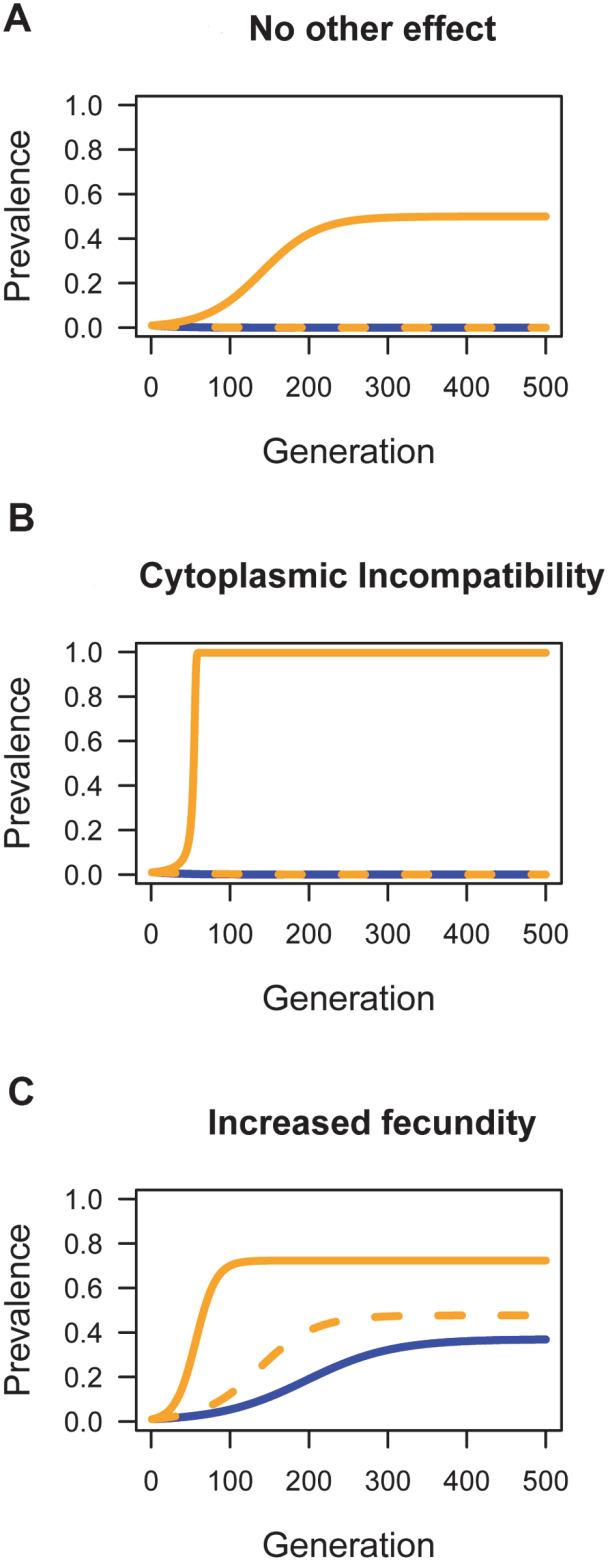
The effect of horizontal transmission on the spread of *Wolbachia* through populations. Simulations were performed to explore the effect of horizontal transmission on the prevalence of *Wolbachia*. The blue lines are where there was no horizontal transmission (*w* = 0), the dashed orange line was a low rate (*w* = 0.01), and the solid orange line a moderate rate of horizontal transmission (*w* = 0.06). A: The effect of horizontal transmission when there is not effect of *Wolbachia* on host fitness and no reproductive manipulation (*H* = 1, *F* = 1). B: *Wolbachia* induces cytoplasmic incompatibility (*H* = 0.1, *F* = 1). C: *Wolbachia* carries a fitness benefit (*H* = 1, *F* = 1.05). In all cases the starting prevalence was *p* = 0.01 and the rate of imperfect maternal transmission was *μ* = 0.03.

We first investigated the effect of horizontal transmission in the absence of any reproductive manipulation or effect on host fitness (Fig. 6A). The outcome depends on the rate of horizontal transmission relative to the rate at which the infection is lost due to imperfect vertical transmission. When horizontal transmission is low, the infection will tend to be lost, but moderate rates of parasitism (*w* = 0.06) can result in *Wolbachia* invading and reaching a stable equilibrium frequency. Fixation is prevented by imperfect vertical transmission.

Next, we examined how horizontal transmission will affect a strain of *Wolbachia* that causes the reproductive manipulation cytoplasmic incompatibility (CI; Fig. 6B). Combining CI and horizontal transmission can dramatically alter the outcome, with horizontal transmission allowing the near fixation of a strain that would otherwise be lost. The reason for this is that there is a threshold prevalence that CI strains must exceed in order to invade populations. Horizontal transmission can lower or eliminate this threshold. For the parameters used in Fig. 6B, if the starting prevalence is high, then *Wolbachia* can invade and reach a high prevalence without horizontal transmission.

Finally, we examined how horizontal transmission affects a strain that provides a fitness benefit to the host (Fig. 6C). Fitness benefits alone can allow the bacterium to invade the population as a mutualist and reach an intermediate equilibrium frequency maintained due to imperfect vertical transmission. However, horizontal transmission can accelerate the invasion and increase the equilibrium prevalence.

## Discussion

*Wolbachia* has well-described effects on host physiology and reproduction that increase its prevalence in populations [1,5,36–38]. For instance, cytoplasmic incompatibility, female-biased offspring sex ratios and providing protection against viral infection are potent methods that may increase the frequency of infected relative to uninfected matrilines. These mechanisms all rely on bolstering the vertical transmission of the bacterium. On the other hand our study shows that within populations *Wolbachia* may spread horizontally as well. If horizontal transmission proves to be important in other insects, it is tempting to speculate that it may sometimes explain the observation of *Wolbachia* infections with no known phenotypic effect [39]. Furthermore, parasitoids could also potentially vector *Wolbachia* to novel species, such as the other cryptic species of *B*. *tabaci* which share parasitoids with AsiaII7. This could partly explain the plethora of phylogenetic evidence of discordance between host and symbiont phylogenies [8,11,12,40,41].

Parasitoid-vectored horizontal transmission of *Wolbachia* may be important in natural populations. AsiaII7 is an indigenous cryptic species of *B*. *tabaci* in south China, and we have surveyed field populations of this whitefly species and its parasitoids from 2006–2012. *Wolbachia* was detectable in about 30–40% of wild *E*. sp. nr. *furuhashii* throughout this time (S4 Fig.). Furthermore, most AsiaII7 individuals were *Wolbachia-*infected, and the prevalence increased during this time (S4 Fig.). Numerous factors may have contributed to this increase, including fitness benefits and horizontal transmission (see results).

It has been suggested that horizontal transmission from parasitoids to their hosts would be unlikely as parasitized hosts die [8]. The mode of transmission we have described here relies on the fact that parasitoids do not always kill hosts with which they interact. Given mixed-instar nymphs, *Eretmocerus* parasitoids of whitefly *B*. *tabaci* usually exhibit a clear preference for feeding on 1^st^ and 4^th^ instar nymphs, leaving 2^nd^ and 3^rd^ instars for oviposition, while *Encarsia* parasitoids prefer to feed on 1^st^ and 2^nd^ instars, leaving 3^rd^ and 4^th^ instars for oviposition [42,43]. Our FISH and PCR screening revealed that the ovipositors and mouthparts of parasitoids get contaminated with *Wolbachia* when they probe or feed on infected whitefly nymphs. When these parasitoids probe uninfected whitefly nymphs, about one third survived, and of those 93.8% became infected with *Wolbachia*. Similar to *Eretmocerus emiratus* parasitoids developing in *Rickettsia*-infected *B*. *tabaci* hosts [7], *Wolbachia* failed to penetrate the oocytes of the parasitoid, but this is not required for parasitoids to transmit the bacterium effectively.

Subsequent to horizontal transmission, the bacteria have to be transmitted efficiently from mother to daughter in order to persist in the population. In many such cases infections fail to persist [1,7] or are transmitted with poor fidelity [16,17,23,44,45]. For example, *Wolbachia* was transmitted at a low rate (3.2%) from an infected *Drosophila simulans* host to the parasitoid *Leptopilina boulardi*, and subsequently was lost within four generations [15]. In our experiment, *Wolbachia* persisted at a stable prevalence (85.0–90.0%) for at least five generations in AsiaII7 whitefly, suggesting that the vertical transmission rates were high. Our result support the recent study of Gehrer and Vorburger [25] in which they demonstrated that parasitoids can transfer the bacterial endosymbionts *Hamiltonella defensa* and *Regiella insecticola* by sequentially stabbing infected and uninfected individuals of the black bean aphid *Aphis fabae*. Similar to our results, this established new, heritable infections.

Vectors that transmit infection can either be biological vectors, where the infective agent replicates or develops in the vector, or phoretic (mechanical) vectors where it does not. Three lines of evidence indicated that the parasitoids are acting as phoretic vectors. First, FISH indicates that the *Wolbachia* is only found on the surface of the wasp and does not infect its tissues or ovaries. Second, the infectivity of the wasps rapidly declines after they have become contaminated with *Wolbachia*. Third, the *Wolbachia* infection was routinely monitored in population cages, and wasps were always uninfected unless they were recently exposed to infected AsiaII7 whiteflies. Therefore, it seems most likely that the wasp is acting as little more than a dirty needle. Our FISH experiments revealed that in all the newly infected AsiaII7 individuals, *Wolbachia* had a scattered distribution across tissues, while vertically infected insects tended to have *Wolbachia* largely confined to the ovaries. Therefore we cannot be sure that the confined distribution of *Wolbachia* in whitefly ovaries can also be horizontal transmitted from one individual to another by parasitoids.

Facultative endosymbionts, including *Wolbachia*, can change the fitness or biology of their hosts. For example, facultative symbionts in the pea aphid (*Acyrthosiphon pisum*) can protect their hosts against entomopathogenic fungi and parasitoid wasps, ameliorate the detrimental effects of heat, and influence host plant suitability [1,46–48]; *B*. *tabaci* Mediterranean cryptic species infected with *Wolbachia* showed decreased juvenile development time, increased juvenile survival, increased adult life span and an increased percentage of female progeny [49]. Similarly, our study also showed that *Wolbachia*-infected AsiaII7 survived for longer and developed faster. There was also an increase in the proportion of daughters produced, but our sample size was small and the effect was not statistically significant. *Wolbachia* always had imperfect vertical transmission, with infected females producing uninfected daughters, which may explain why natural populations are a mixture of infected and uninfected individuals.

Our study revealed changes in fitness caused by *Wolbachia* that can potentially increase the rate of population growth of whitefly, leading to more severe damage to crops. Fitness measurements can often prove context-dependent and hard to replicate, so it is important to reproduce our results in the field. Furthermore, our laboratory results should be replicated in case there are unaccounted for differences between our *Wolbachia*-infected and *Wolbachia*-free populations. However, assuming similar effects are found in nature, our results suggest that parasitoids used for biological control could result in unintended negative consequences when the control agent also allows the horizontal transmission of *Wolbachia* between matrilines or even species of pests. With increased chemical resistance of whitefly in many countries, parasitoids have become an important biocontrol agent to manage whitefly infestations, especially in greenhouses. The *B*. *tabaci* species complex is known to be host to at least 56 species of parasitoids, mostly from the genera *Eretmocerus* and *Encarsia* [35], and some of these have been commercially produced and applied. Usually one *B*. *tabaci* cryptic species can be parasitized by dozens of parasitoid species, and one parasitoid species can attack several *B*. *tabaci* cryptic species, or even more distantly related species of whitefly such as the greenhouse whitefly *Trialeurodes vaporariorum*. This may provide many opportunities for endosymbionts to horizontally transmit between different whitefly species, potentially causing the detrimental fitness changes. We propose that this unintended negative impact of parasitoids in pest biological control cannot be ignored.

## Methods

### Survey of *Wolbachia* infection in whitefly and parasitoid

Adult AsiaII7 whiteflies were first collected from variegated laurel plants, *Codiaeum variegatum* (L.), in Guangzhou in 2006 [50]. They were then reared on hibiscus in two separate greenhouses (15×7m) in the South China Agricultural University (SCAU). These were covered with PVC film on the top and nylon net (70 mesh) on the sides. Two subcolonies were set up using 5–6 cages in the laboratory, one of which was used for AsiaII7 whitefly rearing only and the other was used for parasitoid *E*. sp. nr. *furuhashii* rearing. In the parasitoid rearing cages, new and clean hibiscus plants were added to replace the old plants when needed. This allows both the *B*. *tabaci* hosts and *Eretmocerus* parasitoids to stably coexist because removing old plants takes the parasitoid pupae away. Both the AsiaII7 whitefly and *Eretmocerus* parasitoids were reared in an insect growth chamber at 26.0±0.5°C, 70–80% relative humidity, 14:10 h (L:D) photoperiod.

AsiaII7 samples were collected three times per year (Apr-May, Aug-Sep, Dec-Jan) from both the two subcolonies between 2007–2012. In each survey, 30–50 samples per plant and 3 plants were collected in each subcolony using an aspirator. All samples were preserved immediately in 95% ethanol. Whitefly species identity was confirmed using mtCOI sequences [51,52]. From each survey, 20 whitefly adults from each subcolony were selected randomly for PCR-based detection of *Wolbachia* following the methods of Ahmed et al. [53]. Briefly, *Wolbachia* was detected using primers for the *wsp*, *ftsZ* and 16S rRNA genes. Both negative controls (ddH_2_O) and a template DNA quality control (primary endosymbiont *Portiera* 16S rRNA gene to indicate the DNA quality of extraction) were included. Results were further confirmed using FISH. Alongside the whitefly collection, 20 individuals of *E*. sp. nr. *furuhashii* adults were also sampled for *Wolbachia* detection with PCR and FISH. To identify the *Wolbachia* strain in parasitoids and whiteflies, we used multi locus strain typing methods described in Baldo et al. [54].

### Establishment of *Wolbachia* infected and uninfected AsiaII7 cultures

To establish *Wolbachia*-positive lines of AsiaII7, male/female pairs from the AsiaII7 rearing cages were allowed to reproduce on hibiscus, one pair per plant. Once the F1 progeny emerged, ten pairs of newly emerged adults from one parent were selected at random. Five pairs were tested for the presence of *Wolbachia* using PCR; the remaining five pairs were caged individually on hibiscus leaves. Any line that contained uninfected individuals was discarded until a line in which all five of the tested pairs were positive for *Wolbachia* was identified. This screening process, based on five pairs being selected from each generation, was continued for 10 generations prior to use in experiments. After the initial selection of the line, all individuals screened were positive for *Wolbachia*.

To establish an outbred population, which we used for measuring fitness and in other experiments, we mixed together a large number of these lines. In total 50 to 80 pairs of parents were randomly selected, and all the validated *Wolbachia* positive couples were released into a rearing cage to reproduce. The progeny that emerged were used for the fitness measurements below.

To establish a pure culture of *Wolbachia*-free whitefly, the process followed that used to establish the infected line except that here the initial line was the one where all five pairs tested negative. The infected and uninfected populations were sampled from the same original greenhouse population. Both the infected and uninfected lines of AsiaII7 were then kept in rearing cages (60×60×60 cm) in separated air-conditioned insect growth chamber at 26.0±0.5°C, 70–80% relative humidity, 14:10 (L:D) photoperiod and light intensity of approximately 3000 Lux. *Wolbachia*-infection status in the cages was monitored on a monthly basis by PCR.

### *Wolbachia* horizontal transmission between AsiaII7 nymphs

In the laboratory, we examined horizontal transmission of *Wolbachia* from the infected AsiaII7 nymphs to the uninfected nymphs via parasitoids. About 10 pairs of uninfected AsiaII7 adults were released into a leaf cage to reproduce on hibiscus for 48 h. Progeny from these adults developed to 2^nd^-3^rd^ instar, at which point all but 60 nymphs were removed. Before the experiment, three mated parasitoid females of *E*. sp. nr. *furuhashii* (2-day age) were first introduced into a leaf cage in which there are 2^nd^-3^rd^ instar *Wolbachia*-positive AsiaII7 nymphs for 2h of feeding and oviposition. After that, the parasitoids were transferred into the leaf cages with the 60 *Wolbachia*-free 2^nd^-3^rd^ instar nymphs. The parasitoids were allowed to feed and oviposit for another 2 h, oviposition behaviors were observed using a binocular dissecting microscope, and nymphs visited by the wasp were marked with indelible ink. We used 240 nymphs of AsiaII7 in total (60 in each repeat), of which 106 were visited by parasitoids. Seven days later, the number of nymphs that were parasitized (larval parasitoids are visible in whitefly hosts), the number of nymphs that were subjected to the insertion of an ovipositor but survived, and nymphs that were parasitized but died were recorded. Meanwhile, the emerged whiteflies and parasitoids were both collected for *Wolbachia* detection by PCR following the methods of Ahmed et al. [53]. All the transmission experiments were done at 26.0±0.5°C, 70–80% relative humidity, 14:10 h of L:D photoperiod, and the four repeats were replicated contemporaneously.

### FISH detection of *Wolbachia* in whitefly and parasitoid

The FISH procedure to detect *Wolbachia* in whitefly AsiaII7 and parasitoid *E*. sp. nr. *furuhashii* followed the method of Xue et al. [49] and Sakurai et al. [55]. About twenty *Eretmocerus* female adults (2-day old) were recaptured 24–48 h after they had been released to attack abundant *Wolbachia* positive AsiaII7 nymphs in a leaf cage. For the whitefly samples, we investigated forty 3^rd^ instar AsiaII7 nymphs from the *Wolbachia*-infected donor PR cages (thirty were finally FISH photographed successfully), a similar number of the *Wolbachia* newly infected AsiaII7 nymphs via parasitoid and their F1 generation offspring were randomly selected for FISH detection respectively.

Two 5’ rhodamine labeled *Wolbachia* probes (targeted 16S rRNA of *Wolbachia*) were used to increase the signal: W2: 5’-CTTCTGTGAGTACCGTCATTATC-3’ and W3: 5’-TCCTCTATCCTCTTTCAATC-3’. Stained samples were washed thoroughly twice in the same buffer at 48°C for 20 min. All the samples were observed using an inverted fluorescence microscope (Nikon Eclipse Ti-U). Specificity of the detection was confirmed using the uninfected whiteflies as controls.

In order to quantify the increase of *Wolbachia* after it was transmitted into *Wolbachia* negative AsiaII7 whiteflies, 60 early 3^rd^ instar AsiaII7 nymphs that have been fed or probed by those *Wolbachia*-carrying *Eretmocerus* parasitoids were screened out and divided into three groups. Then the three groups were used for *Wolbachia* qRT-PCR detection 24–72 h after they were fed or probed by parasitoids using the primers and protocols described in Xue et al. [49].

### Stable infection in whitefly

In order to know the stability of *Wolbachia* infection in the newly infected AsiaII7 *B*. *tabaci*, a pair of newly infected adults of whitefly were randomly selected and introduced into a leaf cage for 48 ho. Eggs were allowed to develop to F1 adults, at which point 10 of them were randomly selected and tested for *Wolbachia*. Then another pair of F1 generation adults were randomly selected again and released into a new leaf cage to reproduce F2 generation, among which 10 individuals of F2 adults were selected randomly for PCR detection. This procedure was repeated through to the F5 and only female adults were selected for PCR detection in each generation. A control experiment was conducted in which uninfected AsiaII7 whitefly was reared and checked for *Wolbachia* infection from F1 to F5 generations in separated cages. Both the *Wolbachia* infected and uninfected whitefly lines were reared in the insect growth chamber and four replicates were conducted in each generation contemporaneously.

### Temporary infection in parasitoids

After *E*. sp. nr. *furuhashii* fed or penetrated whitefly nymphs, FISH showed that *Wolbachia* was found in their mouthparts and ovipositors, but not in their ovaries. We therefore hypothesized that these wasps were contaminated temporarily by *Wolbachia* and can transfer this endosymbiont to other hosts by feeding or oviposition. To confirm this hypothesis, 70 to 80 mated females of *E*. sp. nr. *furuhashii* were divided into seven groups and released into seven leaf cages with abundant 2^nd^-3^rd^ instar *Wolbachia-*positive AsiaII7 nymphs. Parasitoids were allowed to oviposit or feed on the whitefly hosts for 48 h, then all of them were recaptured, introduced into seven Petri dishes (9 cm diameter), and fed with 10% honey water. Hereafter one group of the female parasitoids per day was used to detect the presence of *Wolbachia* DNA by PCR according to the method of Ahmed et al. [43]. To extract DNA, 10 parasitoids were homogenized in one tube due to the tiny titer of *Wolbachia*.

### Changes in transmissibility of *Wolbachia*

In order to confirm the time that *Wolbachia* on the mouthparts and ovipositors of *Eretmocerus* wasps is alive and transmittable, five two-day-old female wasps were collected after they parasitized several *Wolbachia*-positive AsiaII7 nymphs. They were then released into a 5 cm diameter Petri dish, and fed with 10% honey solution but not given hosts to parasitize. After 24, 48, 72 and 96 h, these parasitoids were released into leaf cages to parasitize 2^nd^-3^rd^ instar nymphs of *Wolbachia*-negative AisaII7. After 4–6 days, DNA was extracted from individual whitefly adults that emerged, and these samples were tested for the presence of *Wolbachia* by PCR. The experiment at each time point (24–96 h) was repeated four times contemporaneously.

### Fitness effects of *Wolbachia* on AsiaII7 whitefly

To compare the biology of *Wolbachia* uninfected and infected AsiaII7 populations, their development time, immature survivorship, sex ratio, fecundity and adult longevity were investigated according to Qiu et al. [34]. Briefly, 10 pairs of newly emerged AsiaII7 adults were selected at random from the uninfected or infected populations (see above for how these were established). Each pair was introduced into a leaf cage on a hibiscus plant to lay eggs for 48 h (10 leaf cages in each replicate of the experiment), after which all but 20 eggs per leaf cage were removed. The twenty F1 eggs were allowed to develop to adults in the leaf cage, and their emergence were checked daily until all nymphs completed their development. The developmental time of one randomly-selected individual in each leaf cage was recorded (in total 10 individuals were selected in each replicate).

Meanwhile, a pair of *Wolbachia* positive and negative F1 emerged adults (0–12 h age, which is before whitefly begin laying eggs) were randomly selected from each of the 10 leaf cages, and each pair was introduced into a new leaf cage on a hibiscus plant for reproduction. Their eggs were counted daily until the female died, and newly emerged males were supplemented if the original male died before the experiment ended. The fecundity and longevity of F1 generation adults was recorded. In addition, about 100 F1 adults were randomly selected and their sex ratio recorded. All the experiments were performed in the insect growth chamber (26.0±0.5°C, 70–80% relative humidity, 14:10 h of L:D photoperiod). Care was taken to select uniform hibiscus plants. We replicated the experiments measuring fitness trait four times contemporaneously (i.e. there were four replicates of the *Wolbachia-*infected and four replicates of the *Wolbachia*-free treatments).

### Data analysis

The data on the fitness effects of *Wolbachia* was analysed using a series of statistical models. In all the experiments measuring fitness components, both the *Wolbachia*-infected and *Wolbachia-*free treatments were replicated four times, with 10 pairs per replicate (80 pairs in total). The 8 replicates (4 *Wolbachia-*infected and 4 *Wolbachia*-free) were performed contemporaneously. In all cases the model included the full replicate structure of the experiment and no model simplification was performed.

The data on adult longevity consisted of observations on the lifespan of a single adult offspring from each pair (10 observations per replicate). Each adult was observed until it died, so there is no right censoring of the data. We analyzed this data using a Cox proportional hazard model, which included a fixed effect of *Wolbachia* infection status and a random effect of replicate (also see S2 Table). The model was fitted using the function coxme in R.

The data on fecundity consisted of a count of the number of eggs laid by each pair (10 egg counts per replicate). The data on immature development consisted of the development time of a single nymph from each pair (10 nymphs per replicate). We treated both of these datasets as Gaussian and analyzed them using a general linear model. The model included a fixed effect of *Wolbachia* infection status, a random effect of replicate and a residual that accounts for variation between pairs within each replicate.

The data on immature survival consisted of counts of nymphs from each pair that died or survived (10 ratios of dead: alive nymphs per replicate). The data on sex ratio consisted of counts of male and female offspring that were produced by each replicate. Both immature survival and sex ratio were treated as ratios and analysed using generalized linear mixed models with a binomial error structure. The model included a fixed effect of *Wolbachia* infection status, a random effect of replicate for the survival data, and a residual variance to allow for over-dispersion (i.e. variation between pairs within each replicate that was greater than that expected under binomial sampling).

The figures are plotted using parameters estimated from these models, with the exception of adult longevity where mean survival times were estimated for the figure using a model similar to that described above for the fecundity data. For the binomial data, the means and standard errors were back-transformed from the logit scale into proportions for plotting. The model parameters were estimated by maximum likelihood using the R functions lme and glmer in the package lme4 [56].

### Multi locus sequence typing of *Wolbachia* and phylogenetic analysis

Multi locus sequence typing (MLST) was used to identify *Wolbachia* in both AsiaII7 whitefly and *E*. sp. nr. *furuhashii* parasitoids. Five MLST genes, *gatB* (wMel locus WD_0146), *coxA* (WD_0301), *hcpA* (WD_0484), *ftsZ* and *fbpA* (WD_1238), together with the *Wolbachia* surface protein gene (*wsp*) were used for *Wolbachia* strain identification. The process is described in Baldo et al. [54].

Concatenated sequences of the seven most closely related *Wolbachia* sequence types (STs) associated with different host species were selected from the MLST database and used for comparisons with the STs isolated from other whitefly cryptic species, AsiaII7 and the parasitoid, *E*. sp. nr. *furuhashii*. Three STs from supergroup A, D and F *Wolbachia* were used as outgroups. The best model was chosen using the Bayesian Information Criterion (BIC) in MEGA5 [57]. Phylogenetic analysis based on the concatenated data of the five *Wolbachia* MLST loci (2079 bp) was undertaken using maximum parsimony (MP) and maximum likelihood in MEGA5 [57] and PAUP version 4.0b [58]. T92+G model was used during ML tree constructions.

### Simulations of *Wolbachia* dynamics

To understand how horizontal transmission can affect the dynamics of *Wolbachia* within insect populations we adapted the model of Hoffman et al. [59], who provide a full explanation of the model, the parameters and its assumptions. This model assumes discrete generations. Infected females produce a fraction μ of uninfected ova. The fecundity of *Wolbachia-*infected females relative to *Wolbachia-*free females is denoted by *F*. When the strain induced cytoplasmic incompatibility, the relative hatch rate of incompatible crosses is *H*. Let *s*_*h*_ = 1-*H* and *s*_*f*_ = 1-*F*. The prevalence (proportion of infected adults) at generation *t* is *p*_*t*_. In the absence of horizontal transmission, the prevalence at generation *t+*1 is denoted *k*. Following Hoffmann et al. [59]:

$$k=\frac{p_{t_{}}(1-\mu )(1-s_{f})}{1-s_{f}p_{t}-s_{h}p_{t}(1-p_{t})-\mu s_{h}p_{t}^{2}(1-s_{f})}$$

We then added an extra step in which vectors can transmit *Wolbachia* horizontally. We assume that *Wolbachia* in newly infected whitefly has the same effect on female fecundity and cytoplasmic incompatibility as in stably infected lines. We also assume that the rate of hosts being visited by vectors is constant across generations, that wasps visit whitefly randomly with respect to their *Wolbachia* infection status, and that a contaminated wasp can only infect the next whitefly that it visits (i.e. *Wolbachia* is lost by probing an uninfected host). Male and female whiteflies are assumed to be equally likely to be visited by wasps and to transmit or be infected by *Wolbachia*. For simplicity, we also modelled the effects of a vector that never kills its host, although unpublished simulations suggest this produces similar results to a parasitoid that can kill its hosts. We define a new parameter *w* as the number of new cases generated by horizontal transmission from a single infected host in an otherwise uninfected population. It is possible that females that are infected by horizontal transmission may transmit *Wolbachia* at a reduced rate. If this is the case, then *w* can be considered as the number of product of the number of new cases generated by horizontal transmission and the proportionate reduction in the transmission rate in these females. Therefore, the prevalence at generation *t+*1 is given by:

$$P_{t+}{}_{1}=k+wk\left(1-k\right).$$

The value of *w* will be determined by factors including the frequency with which vectors visit hosts relative to the time that the contaminated vectors remain infectious and the probability that a contaminated wasp infects an uninfected whitefly.

## Supporting Information

S1 Table Details of the MLST gene sequences of Wolbachia in AsiaII7 whitefly and *Eretmocerus* sp. nr. *furuhashii* parasitoids. (DOC)

S2 Table The effect of experimental replicate on fitness traits. This table reports the effect sizes and significance of the random effect of replicate and the residual. Note that the variances are estimated from 8 observations (4 *Wolbachia-*infected and 4 *Wolbachia-*free) and are therefore poorly estimated. The significance of random effects was assessed using likelihood ratio tests.
(DOC)

S1 Fig The prevalence of Wolbachia in lab populations of Bemisia tabaci AsiaII7 and Eretmocerus parasitoids during 2007–2012. The prevalence of *Wolbachia* in a single cage of whitefly containing parasitoids (red points are the whitefly and grey points the wasps), and a single cage of whitefly without parasitoids (black). Each point is from 20 individuals. The lines are predicted values from a logistic regression that have been back transformed from a logit scale. The whitefly in the cage containing parasitoids have a significantly greater prevalence of *Wolbachia* than those from the cage without parasitoid (logistic regression: *z* = 2.06, *P* = 0.04), and there is a significant increase in prevalence through time in all three datasets (logistic regression, main effect of date: *z* = 2.86, *P* = 0.004). There is no significant difference in the rate at which the prevalence increases among the three groups.
(TIF)

S2 Fig The detection of Wolbachia in the newly infected AsiaII7 Bemisia tabaci nymphs with qRT-PCR. There is a continuously increase of *Wolbachia* in the 24–72 h after the whitefly was infected by a contaminated parasitoid. The Y-axis shows the quantity of *Wolbachia* (value = 2^-Ct^) in each treatment. NegCon: negative control, *Wolbachia*-negative AsiaII7 nymph; PosCon: positive control, donor *Wolbachia*-positive AsiaII7 nymph.
(TIF)

S3 Fig Maximum Likelihood phylogeny of Wolbachia strains based on five MLST genes. The bootstrap support (*1000* replicates) is indicated on the branch leading to each node. Strain types (ST), strain identities (ID), host species, *Wolbachia* supergroups of each strain are shown in the tree according to the information available at the MLST website.
(TIF)

S4 Fig The prevalence of Wolbachia in the Bemisia tabaci AsiaII7 whitefly and Eretmocerus parasitoid field populations (Guangzhou) during 2006–2012. Points are means with standard errors.
(TIF)
