# Supplementary material for: The Intracellular Bacterium Wolbachia Uses Parasitoid Wasps as Phoretic Vectors for Efficient Horizontal Transmission
Source: PLoS Pathog. 2015 Feb 12;11(2):e1004672. doi: 10.1371/journal.ppat.1004672 (PMC4347858; doi:10.1371/journal.ppat.1004672)
Supplement: S1 Table — (DOC) [file ppat.1004672.s001.doc]

**Supplementary files**

**Table S1**

| Gene | Whitefly, *Bemisia tabaci* (AsiaII7) | | *Eretmocerus* sp. nr. *furuhashii* | |
| --- | --- | --- | --- | --- |
|  | NCBI accession number | MLST Alleles ID | NCBI accession number | MLST Alleles ID |
| wsp | KJ600634 |  | KJ600633 |  |
| ftsZ | KJ600641 | 105 | KJ600642 | 105 |
| coxA | KJ600647 | 88 | KJ600648 | 88 |
| hcpA | KJ600637 | 106 | KJ600636 | 106 |
| gatB | KJ600639 | 105 | KJ600640 | 105 |
| fbpA | KJ600644 | 387 | KJ600645 | 387 |
| MLST strain ID |  | 618 |  | 619 |
| MLST strain type |  | 388 |  | 388 |
