# Supplementary material for: The Intracellular Bacterium Wolbachia Uses Parasitoid Wasps as Phoretic Vectors for Efficient Horizontal Transmission
Source: PLoS Pathog. 2015 Feb 12;11(2):e1004672. doi: 10.1371/journal.ppat.1004672 (PMC4347858; doi:10.1371/journal.ppat.1004672)
Supplement: S2 Table — This table reports the effect sizes and significance of the random effect of replicate and the residual. Note that the variances are estimated from 8 observations (4 Wolbachia-infected and 4 Wolbachia-free) and are therefore poorly estimated. The significance of random effects was assessed using likelihood ratio tests. (DOC) [file ppat.1004672.s002.doc]

**Table S2**

| Trait | Parameter | Variance | Units | Chi-squared statistic | Degrees of freedom | *P* |
| --- | --- | --- | --- | --- | --- | --- |
|  |  |  |  |  |  |  |
| Sex ratio | Residual | 0.015 | logits | - | - | - |
|  |  |  |  |  |  |  |
| Development time | Replicate | 0.10 | Days | 0.005 | 1 | 0.94 |
| Development time | Residual | 1.52 | Days | - | - | - |
|  |  |  |  |  |  |  |
| Immature survival | Replicate | 0 | logits | 0 | 1 | 1 |
| Immature survival | Residual | 0 | logits | - | - | - |
|  |  |  |  |  |  |  |
| Fecundity | Replicate | 231.30 | Number eggs | 7.14 | 1 | 0.008 |
| Fecundity | Residual | 831.73 | Number eggs | - | - | - |
|  |  |  |  |  |  |  |
| Adult survival | Replicate | 0.074 | Hazard ratio | 1.29 | 1 | 0.26 |
