# Supplementary figures and images for: The Intracellular Bacterium Wolbachia Uses Parasitoid Wasps as Phoretic Vectors for Efficient Horizontal Transmission
Source: PLoS Pathog. 2015 Feb 12;11(2):e1004672. doi: 10.1371/journal.ppat.1004672 (PMC4347858; doi:10.1371/journal.ppat.1004672)

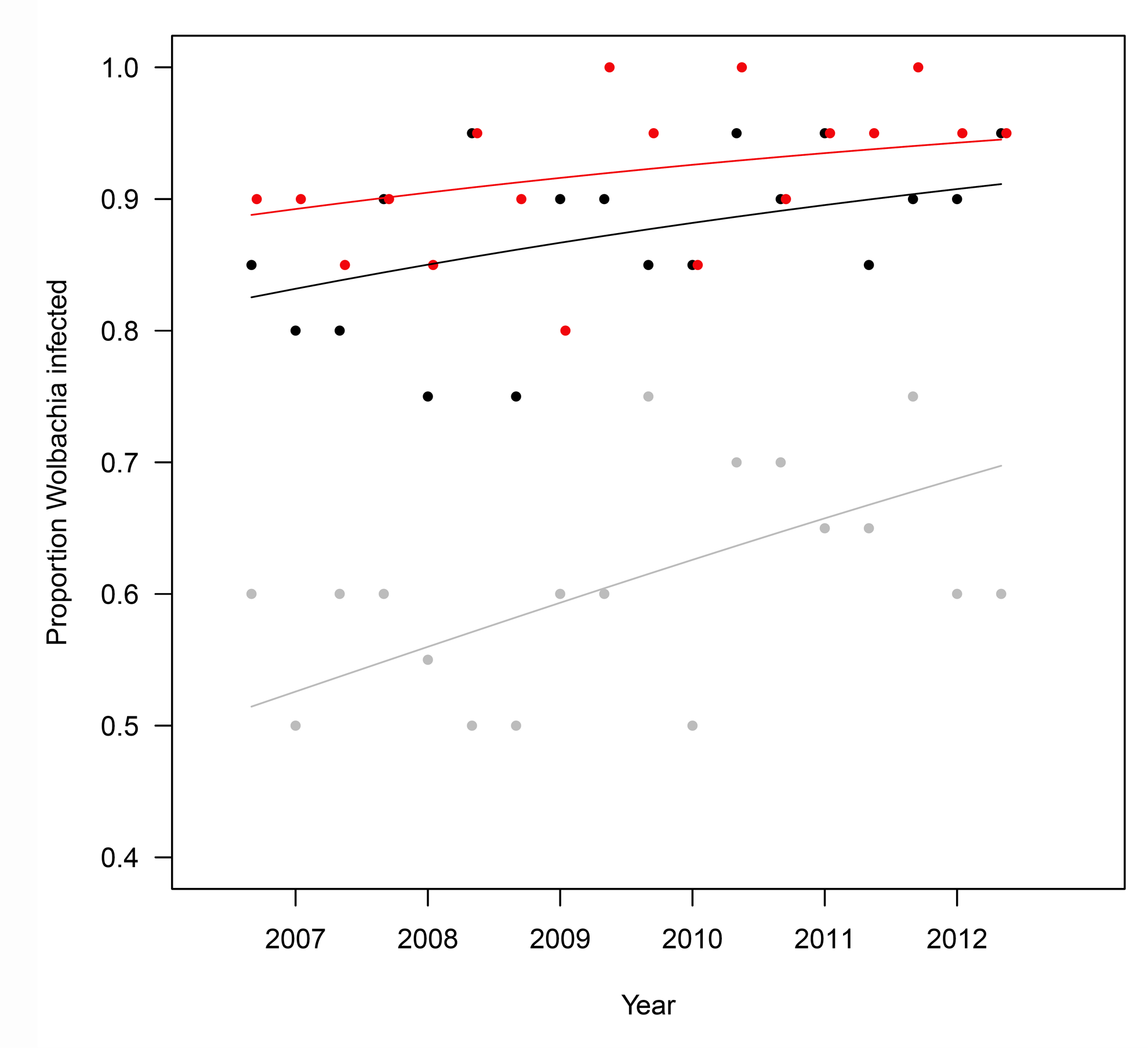

Supplement: S1 Fig — The prevalence of Wolbachia in a single cage of whitefly containing parasitoids (red points are the whitefly and grey points the wasps), and a single cage of whitefly without parasitoids (black). Each point is from 20 individuals. The lines are predicted values from a logistic regression that have been back transformed from a logit scale. The whitefly in the cage containing parasitoids have a significantly greater prevalence of Wolbachia than those from the cage without parasitoid (logistic regression: z = 2.06, P = 0.04), and there is a significant increase in prevalence through time in all three datasets (logistic regression, main effect of date: z = 2.86, P = 0.004). There is no significant difference in the rate at which the prevalence increases among the three groups. (TIF) [file ppat.1004672.s003.tif]

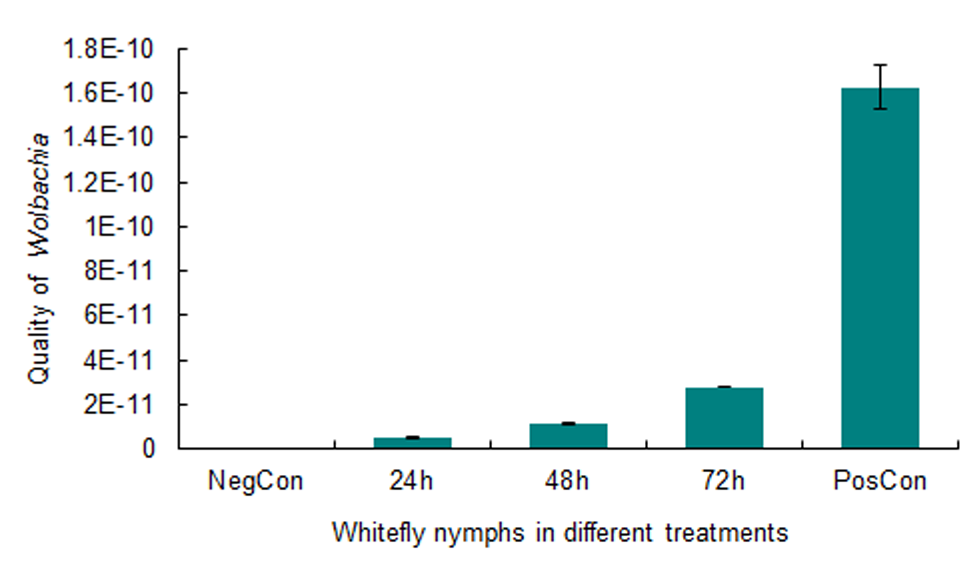

Supplement: S2 Fig — There is a continuously increase of Wolbachia in the 24–72 h after the whitefly was infected by a contaminated parasitoid. The Y-axis shows the quantity of Wolbachia (value = 2-Ct) in each treatment. NegCon: negative control, Wolbachia-negative AsiaII7 nymph; PosCon: positive control, donor Wolbachia-positive AsiaII7 nymph. (TIF) [file ppat.1004672.s004.tif]

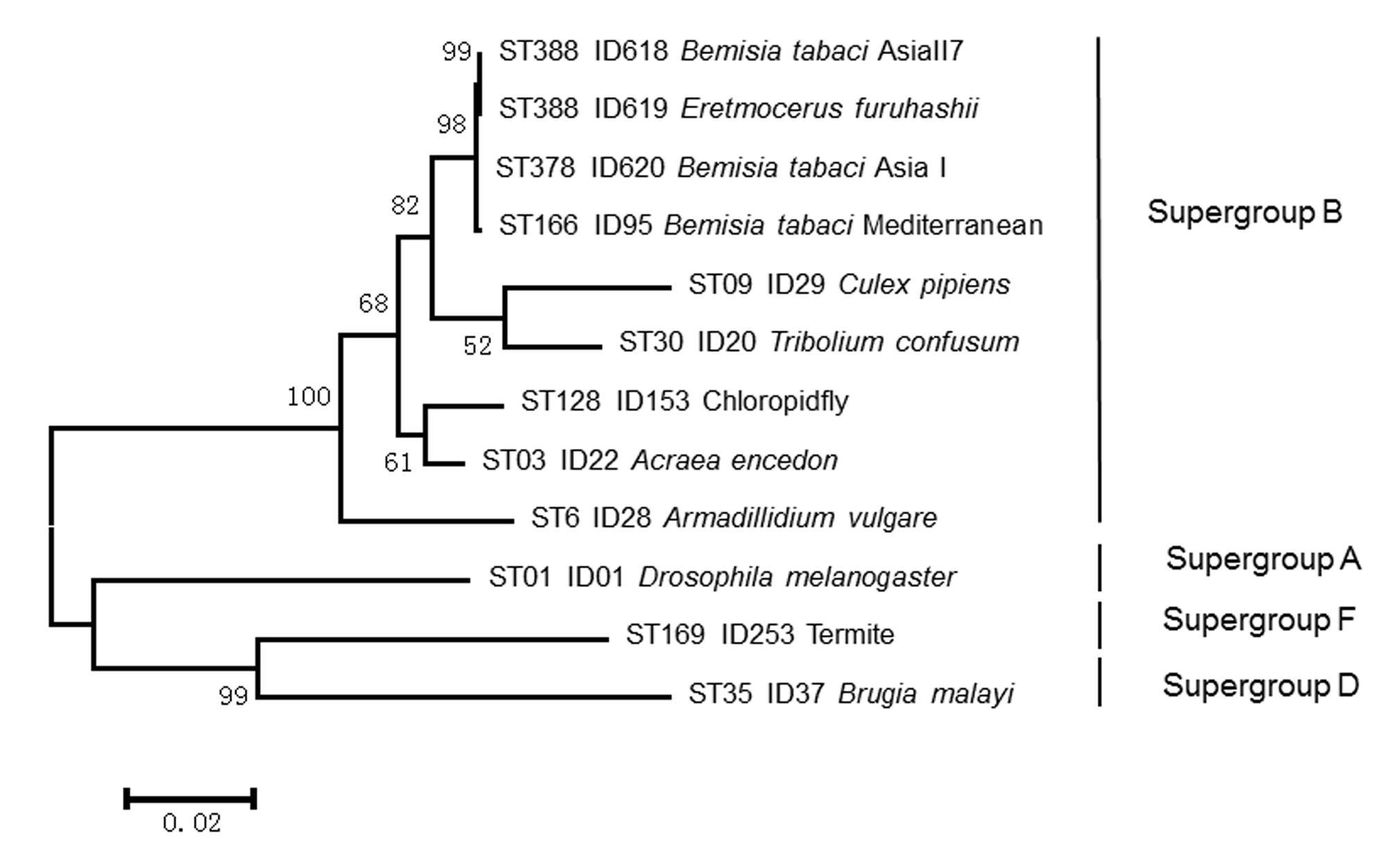

Supplement: S3 Fig — The bootstrap support (1000 replicates) is indicated on the branch leading to each node. Strain types (ST), strain identities (ID), host species, Wolbachia supergroups of each strain are shown in the tree according to the information available at the MLST website. (TIF) [file ppat.1004672.s005.tif]

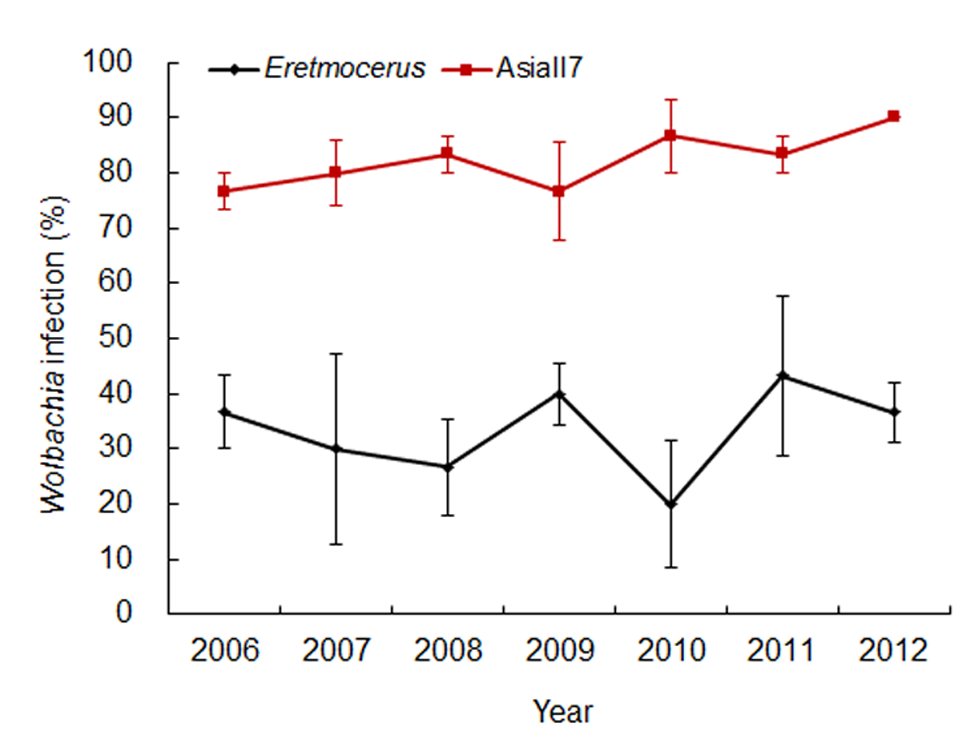

Supplement: S4 Fig — Points are means with standard errors. (TIF) [file ppat.1004672.s006.tif]
